# An RNA-dependent mechanism for transient expression of bacterial translocation filaments

**DOI:** 10.1093/nar/gky096

**Published:** 2018-02-08

**Authors:** Dai Wang, Sean P McAteer, Agata B Wawszczyk, Clark D Russell, Amin Tahoun, Alex Elmi, Scott L Cockroft, David Tollervey, Sander Granneman, Jai J Tree, David L Gally

**Affiliations:** 1 Division of Infection and Immunity, The Roslin Institute and R(D)SVS, University of Edinburgh, Edinburgh EH25 9RG, UK; 2 State Key Laboratory of Molecular Vaccinology and Molecular Diagnostics, School of Public Health, Xiamen University, South Xiangan Rd., Xiangan District, Xiamen, Fujian Province 361102, China; 3 Centre for Synthetic and Systems Biology, University of Edinburgh, Edinburgh EH9 3BF, UK; 4 Faculty of Veterinary Medicine, Kafrelsheikh University, 33516 Kafrel-Sheikh, Egypt; 5 EaStCHEM School of Chemistry, University of Edinburgh, Joseph Black Building, David Brewster Road, Edinburgh EH9 3FJ, UK; 6 Wellcome Centre for Cell Biology, University of Edinburgh, Edinburgh EH9 3BF, UK; 7 School of Biotechnology and Biomolecular Sciences, University of New South Wales, Sydney 2052, NSW, Australia

## Abstract

The prokaryotic RNA chaperone Hfq mediates sRNA–mRNA interactions and plays a significant role in post-transcriptional regulation of the type III secretion (T3S) system produced by a range of *Escherichia coli* pathotypes. UV-crosslinking was used to map Hfq-binding under conditions that promote T3S and multiple interactions were identified within polycistronic transcripts produced from the locus of enterocyte effacement (LEE) that encodes the T3S system. The majority of Hfq binding was within the LEE5 and LEE4 operons, the latter encoding the translocon apparatus (SepL-EspADB) that is positively regulated by the RNA binding protein, CsrA. Using the identified Hfq-binding sites and a series of sRNA deletions, the sRNA Spot42 was shown to directly repress translation of LEE4 at the *sepL* 5′ UTR. *In silico* and *in vivo* analyses of the *sepL* mRNA secondary structure combined with expression studies of truncates indicated that the unbound *sepL* mRNA is translationally inactive. Based on expression studies with site-directed mutants, an OFF-ON-OFF toggle model is proposed that results in transient translation of SepL and EspA filament assembly. Under this model, the nascent mRNA is translationally off, before being activated by CsrA, and then repressed by Hfq and Spot42.

## INTRODUCTION

Enterohaemorrhagic *Escherichia coli* (EHEC) strains can cause life-threatening infections in humans and the main serotype associated with disease in parts of Europe, North America and Japan is O157:H7. Cattle are the primary reservoir for EHEC O157 strains (1) and colonization of cattle or humans is dependent on a type III secretion (T3S) system expressed from the LEE pathogenicity island (2). The T3S system injects multiple effector proteins into host epithelial cells with various functions including cytoskeletal manipulation for intimate bacterial adherence and suppression of host cell inflammatory responses (2,3). Assembly of the T3S system is a staged process requiring the expression of a basal apparatus spanning both bacterial membranes (LEE1–3 operons) before production of the hollow filaments (LEE4 operon) through which effector proteins will be transferred (4), including the translocated intimin receptor (Tir) expressed from LEE5. T3S translocation filaments are produced by both EHEC and enteropathogenic *E. coli* (EPEC) and are composed of EspA (5). These filaments allow secretion of bacterial effector proteins through a pore in the host cell membrane composed of EspD and EspB, all expressed from LEE4 (6,7). Together this structure is known as the translocon and its secretion is dependent on two interacting proteins, SepL and SepD, that govern the switch from translocon assembly to effector protein secretion (8–10).

**Figure 1.**
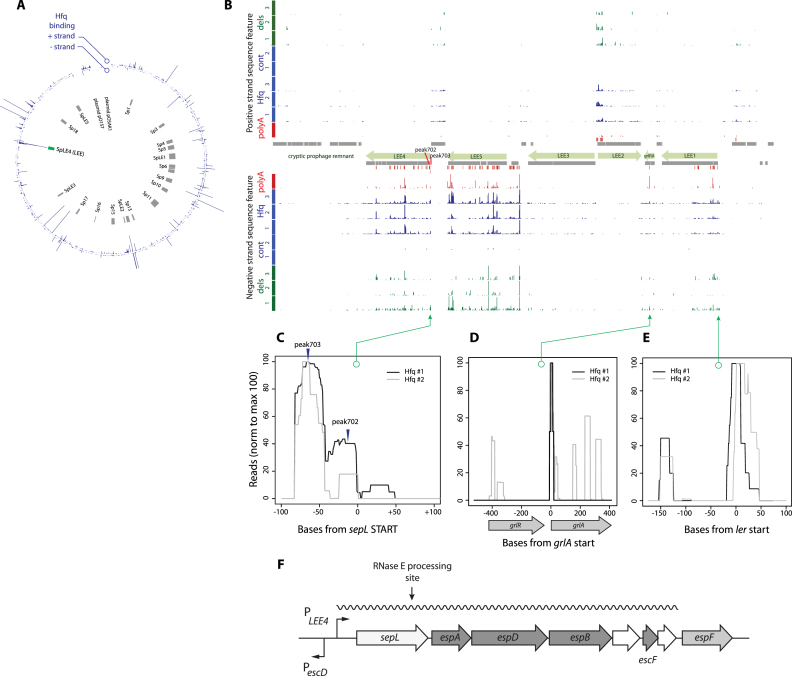
The RNA chaperone, Hfq, binds multiple sites within the polycistronic mRNAs of the LEE. (**A**) The circular plot defines the location of Hfq-associated RNA sequences within the *E. coli* O157 Sakai genome; shown for both strands based on our previous Hfq UV-crosslinking study (Materials and Methods, (31)). The location of integrated and cryptic prophage elements (Sp), and the Locus of Enterocyte Effacement (LEE) are on the inner ring indicated (grey boxes). (**B**) Hfq binding across the LEE. Genomic features within the LEE are shown (centre, grey boxes), including the five polycistronic LEE operons (LEE1–5, green boxes) and *grlRA*. Positive strand features are shown above the genomic features and negative strand below. Hfq binding sites identified by UV-crosslinking (31) are shown in blue for triplicate datasets. From this data, non-genomically encoded oligo(A) tails (indicative of RNA cleavage sites) were also extracted and plotted in red. Replicate control (wild type untagged Hfq) are also shown in blue and represent the background recovery of mRNAs under our experimental conditions. Deletions in sequencing reads are indicative of direct Hfq-RNA contact and are plotted in green for each Hfq UV-crosslinking experiment. (**C**) Hfq binding sites with the 5′ UTR and 5′ end of *sepL* are shown from two rounds of Hfq-CRAC analysis. The mapping indicates two predominant sites of Hfq interaction at peaks 702 and 703 (indicated by arrowheads). (**D**) Hfq binding at the *grlRA* dicistronic mRNA. Replicate Hfq UV-crosslinking experiments are presented. (**E**) Hfq-binding to the 5′ UTR and first 100nt of the master regulator of the LEE, *ler*. Replicate Hfq UV-crosslinking experiments are shown. (F) Diagram of the LEE4 operon. The operons in the Locus of Enterocyte Effacement (LEE) are ordered LEE1, 2, 3, 5, 4 (opposite direction to that shown in panel B) and LEE4 is positioned after LEE5 and the *escD* promotor (shown). The operon extends from *sepL*, through the translocon-encoding genes, *espADB*, and three genes including *escF* that encodes a needle structure over which the EspA filament is assembled. The polycistronic mRNA is expressed from a promoter (P*_LEE4_*) in front of *sepL*. The LEE4 mRNA is processed by an RNaseE site in the 3′ end of *sepL* (29).

The assembly and function of complex multi-component organelles, such as T3S systems, requires hierarchial control at the transcriptional, translational and post translational levels (4,11,12). Translational control by sRNAs facilitates rapid regulatory responses in bacteria (13–16) and there is increasing evidence for the role of sRNAs in the regulation of virulence factors including T3S systems (17–21). Interactions between mRNAs by sRNAs are often catalysed by Hfq which forms doughnut shaped hexamers that guide sRNA–mRNA interactions. Hfq controls virulence factor expression in a number of different pathogens via sRNA interactions (22). In particular, Hfq is implicated in the regulation of T3S in *E. coli* via expression of the regulatory proteins Ler (LEE1) and GrlA (located between LEE1 and LEE2) (23,24). We previously reported separate post-transcriptional regulation of the LEE4 and LEE5 operons (25,26) and proposed a ‘checkpoint’ in secretion system assembly between the basal apparatus and the translocon filament (4,25). Bhatt et el (27) demonstrated that the regulatory protein CsrA (Carbon Storage regulator A) which acts primarily on mRNA transcripts (28), activates T3S since a *csrA* mutant exhibits reduced secretion of EspADB and Tir. CsrA was shown to bind to the LEE4 transcript, acting at two predicted sites in the *sepL* 5′ UTR (27). CsrA also has a repressive effect when over-expressed due to negative regulation of *grlRA*, indicating that physiological levels of CsrA are required for normal control. Once transcribed, the LEE4 transcript is then processed by RNase E towards the 3′ end of *sepL*, which presumably prevents further SepL production and may have consequences for *espADB* transcript translation (29). While *sepL* and *espADB* are initially transcribed on the same transcript (Figure 1F), there is processing of this transcript (29), and *sepL* mutations can be complemented *in trans* (10,30). In the absence of *sepL*, EHEC has a hypersecretion phenotype consistent with the SepL–SepD complex acting as a gate to control translocon filament production. EspA is still produced inside the bacterium in the absence of *sepL* indicating its main role is to govern assembly of the filaments and not regulate their expression (8,10,30).

We previously employed UV cross-linking and high throughput sequencing of cDNAs (CRAC) to define Hfq-RNA interactions (31). Here, we investigated the role of Hfq in the regulation of translocon expression in *E. coli* O157. CRAC identified LEE mRNA sequences that interact with Hfq to control the expression of T3S components. Using our Hfq-binding data to reduce the sequence space for *in silico* predictions of sRNA–mRNA interactions, we constructed a series of sRNA deletions to determine which sRNAs control translation of *sepL* and T3S secretion more broadly. We show that *sepL* is repressed by Spot42, a small RNA that is highly expressed under the T3S-permissive conditions used. The primary Hfq binding site overlaps a sequence identified for CsrA activation of translation and we provide evidence that the nascent folded *sepL* transcript would be translationally inactive. The interactions identified in our study provide a model for transient translation of protein expression, an OFF–ON–OFF toggle switch, to produce a short burst of SepL expression leading to EspA filament production.

## MATERIALS AND METHODS

### Bacterial strains and plasmids

The strains and plasmids used in this study are described in Tables 1 and 2 respectively. Two strains were studied as they differ markedly in their level of T3S. *Escherichia coli* O157 strain Sakai is a well annotated strain that has a relatively low level of secretion under laboratory conditions and for which a Shiga toxin negative variant is available (Table 1). *Escherichia coli* O157 ZAP193 (NCTC12900, Table 1) is a Shiga toxin negative strain that has a high secretion level compared with strain Sakai. In order to further investigate the role of sRNAs in EHEC O157:H7, sRNA mutants were constructed using allelic exchange with pIB307 as described previously (32). In brief, primer pairs (Table 3) were used to amplify flanking regions of each sRNA and the PCR products were cloned into pIB307 (Table 2). A tetracycline resistance gene from pTOF61 (Table 2) was then cloned using *Not*I restriction sites into the constructs containing sRNA flanking regions, except for pDW-fSft where the *sacB/kan* cassette from pDG028 was used (Table 2). These constructs were then transformed into both *E. coli* O157 str. ZAP193 and *E. coli* O157 strain Sakai *stx-*and allelic exchanges carried out as previously described (32) to generate the sRNA deletion strains (Table 1). The final plasmid constructs (Table 2) were sequenced prior to the deletion exchange and each deletion confirmed by PCR analysis.

**Table 1.** Bacterial strains, media, plasmids and primers used in this study

Strains	Details/reference	Plasmids used for construction (if required)
ZAP193	*E. coli* O157:H7 *stx-*, NCTC 12900	
Sakai *stx-*	Dahan *et al.* (60)	
Sakai s*tx-* Hfq-HTF	Tree *et al.* (31)	
ZAP1419	ZAP193: *micF*-	pDW-mFft then pDW-mFf2
ZAP1420	ZAP193: *micA*-	pDW-mAft then pDW-mAf2
ZAP1421	ZAP193: *rybB*-	pDW-bBft then pDW-bBf2
ZAP1422	ZAP193: *ryhB*-	pDW-hBft then pDW-hBf2
ZAP1423	ZAP193: *omrA*-	pDW-oAft then pDW-oAf2
ZAP1424	ZAP193: *omrB*-	pDW-oBft then pDW-oBf2
ZAP1425	ZAP193: *spf*-	pDW-sfft then pDW-sff2
ZAP1426	ZAP193: *fnrS*-	pDW-fSft then pDW-fSf2
ZAP1427	ZAP193: *mcaS*-	pDW-mSft then pDW-mSf2
ZAP1428	ZAP193: *rprA*-	pDW-rAft then pDW-rAf2
ZAP1429	Sakai: *stx*-, *micF*-	pDW-mFft then pDW-mFf2
ZAP1430	Sakai: *stx*-, *micA*-	pDW-mAft then pDW-mAf2
ZAP1431	Sakai: *stx*-, *rybB*-	pDW-bBft then pDW-bBf2
ZAP1432	Sakai: *stx*-, *ryhB*-	pDW-hBft then pDW-hBf2
ZAP1433	Sakai: *stx*-, *omrA*-	pDW-oAft then pDW-oAf2
ZAP1434	Sakai: *stx*-, *omrB*-	pDW-oBft then pDW-oBf2
ZAP1435	Sakai: *stx*-, *spf*-	pDW-sfft then pDW-sff2
ZAP1436	Sakai: *stx*-, *fnrS*-	pDW-fSft then pDW-fSf2
ZAP1437	Sakai: *stx*-, *mcaS*-	pDW-mSft then pDW-mSf2
ZAP1438	Sakai: *stx*-, *rprA*-	pDW-rAft then pDW-rAf2
ZAP1415	Sakai:*spf*:tet	pDW-sfft
ZAP1772	Sakai: *spf* (WT)	pIB-spf
ZAP1773	Sakai: *spf* (S1)	pIB-spf-S1
ZAP1774	Sakai: *spf* (S2)	pIB-spf-S2

**Table 2.** Plasmids used in this study

Plasmids	Details
pIB307	pMAK705-based vector for allelic exchange; temperature-sensitive replicon (61)
pDW-mAf2	pIB307 containing upstream and downstream flanking regions of *micA*
pDW-mFf2	pIB307 containing upstream and downstream flanking regions of *micF*
pDW-hBf2	pIB307 containing upstream and downstream flanking regions of *ryhB*
pDW-bBf2	pIB307 containing upstream and downstream flanking regions of rybB
pDW-sff2	pIB307 containing upstream and downstream flanking regions of *spf*
pDW-oAf2	pIB307 containing upstream and downstream flanking regions of *omrA*
pDW-oBf2	pIB307 containing upstream and downstream flanking regions of *omrB*
pDW-rAf2	pIB307 containing upstream and downstream flanking regions of *rprA*
pDW-fSf2	pIB307 containing upstream and downstream flanking regions of *fnrS*
pDW-mSf2	pIB307 containing upstream and downstream flanking regions of *mcaS*
pDW-mAft	pDW-mAf2 + *tet* cassette from pTOF61
pDW-mFft	pDW-mFf2 + *tet* cassette from pTOF61
pDW-hBft	pDW-hBf2 + *tet* cassette from pTOF61
pDW-bBft	pDW-bBf2 + *tet* cassette from pTOF61
pDW-sfft	pDW-sff2 + *tet* cassette from pTOF61
pDW-oAft	pDW-oAf2 + *tet* cassette from pTOF61
pDW-oBft	pDW-oBf2 + *tet* cassette from pTOF61
pDW-rAft	pDW-rAf2 + *tet* cassette from pTOF61
pDW-fSft	pDW-fSf2 + *sacB/kan* cassette from pDG028
pDW-mSft	pDW-mSf2 + *tet* cassette from pTOF61
pTOF61	A plasmid derived from pTOF1 (62) has TcR cloned into the SmaI site
pDG028	Low-copy-number vector containing *sacB/kan* cassette (25)
pDW6	pAJR70 digested with *Bam*HI/*Kpn*I; whole *sepL* gene with its own promoter amplified from ZAP193 and inserted (10)
pDW-LEE1	Encodes full length Ler fusion to GFP expressed from WT promoter (33)
pDW-LEE5	Encodes full length Tir fusion to GFP expressed from WT promoter (33)
pDW26	First 51bp of sepL cloned in frame to egfp in pAJR70 (10)
pDW6-S1	Mutation of *sepL* 5′UTR with S1 primers
pDW6-S2	Mutation of *sepL* 5′UTR with S2 primers
pDW6-S3	Mutation of *sepL* 5′UTR with S3 primers
pDW6-C1	Mutation of *sepL* 5′UTR with C1 primers
pDW6-C2	Mutation of *sepL* 5′UTR with C2 primers
pDW6-C3	Mutation of *sepL* 5′UTR with C3 primers
pDW6-C4	Mutation of *sepL* 5′UTR with C4 primers
pDW6-C5	Mutation of *sepL* 5′UTR with C5 primers
pDW6-H1	Mutation of *sepL* 5′UTR with H1 primers
pIB-spf	pIB307 containing spf plus upstream and downstream flanking regions
pIB-spf-S1	pIB-spf mutated with S1 primers
pIB-spf-S2	pIB-spf mutated with S2 primers

**Table 3.** Primers used in this study

Primer	Sequence	Application
micA55	CCCAAGCTTcggcggcatcgagcctc	pDW-mAf2
micA53	GCTCTAGAGCGGCCGC atatactcagactcgcctgg	pDW-mAf2
micA35	GCTCTAGAtctgtcaggcgtgtttttcc	pDW-mAf2
micA33	GGGGTACCtcgcaactactatcgcttcg	pDW-mAf2
micF55	CCCAAGCTTcatcagtacgtttggagctg	pDW-mFf2
micF53	GCTCTAGAGCGGCCGCggtagttattctagttgcgag	pDW-mFf2
micF35	GCTCTAGAtacccttctttacacacttttc	pDW-mFf2
micF33	CGGGATCCagccacgaccgttgccag	pDW-mFf2
ryhB55	CCCAAGCTTtcatatgaagaattgaatctgtg	pDW-hBf2
ryhB53	CGGGATCCGCGGCCGCtgatcgcgagacaataataatc	pDW-hBf2
ryhB35	CGGGATCCggtgctggctttttttttgatc	pDW-hBf2
ryhB33	GGGGTACCcggcttcatctcttctctg	pDW-hBf2
mcaS55	GCTCTAGActttcgctggctgaaactatc	pDW-mSf2
mcaS53	CGGGATCCGCGGCCGCcggtgactgtgttataacgg	pDW-mSf2
mcaS35	CGGGATCCgcagttttaactttgcagatag	pDW-mSf2
mcaS33	GGGGTACCcgttcataaattaccctgacac	pDW-mSf2
rybB55	CCCAAGCTTgattctggcaaatccagacg	pDW-bBf2
rybB53	GCTCTAGAGCGGCCGCggcactaattaagactgatgc	pDW-bBf2
rybB35	GCTCTAGAtgcctgaaatttatctactacc	pDW-bBf2
rybB33	CGGGATCCcacttccgcagaaccaaaac	pDW-bBf2
rprA55	GCTCTAGAgccatttccagcggtgac	pDW-rAf2
rprA53	CGGGATCCGCGGCCGCgagctaatagtaggcatacg	pDW-rAf2
rprA35	CGGGATCCaacatttttccgcttcgctac	pDW-rAf2
rprA33	GGGGTACCaatattggctgtcccaggtg	pDW-rAf2
fnrS55	CGAGCTCcgttaaaccgctggaaaatac	pDW-fSf2
fnrS53	TCCCCCCGGGaagagagatattgccctgaatg	pDW-fSf2
fnrS35	GCTCTAGAtgcctgttatttatcagcgtc	pDW-fSf2
fnrS33	GCATTAATcaaaacaccacgcaagagga	pDW-fSf2
omrA55	GCTCTAGAtacccttgcagacgatcttc	pDW-oAf2
omrA53	CGGGATCCGCGGCCGCggatcttgattgtggtctgc	pDW-oAf2
omrA35	CGGGATCCcgcacctaatttactgtcgc	pDW-oAf2
omrA33	GGGGTACCtggttttgatttcagccgcc	pDW-oAf2
omrB55	GCTCTAGAatcattgagaacgcccatcg	pDW-oBf2
omrB53	CGGGATCCGCGGCCGCggatcaccactttagcaacc	pDW-oBf2
omrB35	CGGGATCCtgccggtcatcaatctgtaac	pDW-oBf2
omrB33	GGGGTACCatttgcgataggagtctggg	pDW-oBf2
Spf55	GCTCTAGAgatgatcaacccgaaaaccg	pDW-sff2
Spf53	CGGGATCCGCGGCCGCgcgactaactttactcttttttg	pDW-sff2
Spf35	CGGGATCCttgggcgaaaggaaagatcc	pDW-sff2
Spf33	GGGGTACCcaaacaatcaaccgccatcag	pDW-sff2
C1 SDM FOR	tagagtagaaatttagctgttgtac	pDW6-C1
C2 SDM FOR	tagagtagaaagcaagctgttgtac	pDW6-C2
C3 SDM FOR	tagagtagaaaggcagctgttgtac	pDW6-C3
C1, C2, C3 SDM REV	ttcttagacgatgtaagttcacc	pDW6-C1, pDW6-C2, pDW6-C3
C4 SDM FOR	gtactatttaattttatattcataattaatg	pDW6-C4
C4 SDM REV	tgtacaacagcttcctttctactctattc	pDW6-C4
C5 SDM FOR	ttacgtgagttaaaaatggctaatg	pDW6-C5
C5 SDM REV	tcattaattatgaatat	pDW6-C5
H1 SDM FOR	acatcgtctaagaacggactagaaaggaagctg	pDW6-H1
H1 SDM REV	aagttcaccatattttttctc	pDW6-H1
S1 SDM FOR	ctatttaatggaatattcaAaattaatgattTgtgagtttccaatggc	pDW6-S1
S1 SDM REV	gccattggaaactcacAaatcattaattTtgaatattccattaaatag	pDW6-S1
S2 SDM FOR	ctatttaatggaatattcaATTttaatgaAATcgtgagtttccaatggc	pDW6-S2
S2 SDM REV	gccattggaaactcacgATTtcattaaAATtgaatattccattaaatag	pDW6-S2
S3 SDM FOR	ctatttaatggaatattcatGGtCaatgaCCacgtgagtttccaatggc	pDW6-S3
S3 SDM REV	gccattggaaactcacgtGGtcattGaCCatgaatattccattaaatag	pDW6-S3
spf-S1 SDM FOR	gaccttttacttcacAaatcggatttggctg	pIB-spf-S1
spf-S1 SDM REV	cagccaaatccgattTgtgaagtaaaaggtc	pIB-spf-S1
spf-S2 SDM FOR	accttttacttcacgATTtcggatttggctgaa	pIB-spf-S2
spf-S2 SDM REV	ttcagccaaatccgaAATcgtgaagtaaaaggt	pIB-spf-S2
sepL-full used with below	aaaaggatccgattgaggccttgttcaag	
sepL-ATG	ggggtaccatggtaccggtcgccaccatg	pDW-sepL-ATG
sepL+6	ggggtaccgtaccggtcgccaccatggtg	
sepL+9	ggggtaccccggtcgccaccatggtgagc	
sepL+12	ggggtaccgtcgccaccatggtgagcaag	
sepL+15	ggggtaccgccaccatggtgagcaagggc	
sepL+18	ggggtaccaccatggtgagcaagggcgag	
sepL+27	ggggtaccagcaagggcgaggagctgttc	
sepL+30	ggggtaccaagggcgaggagctgttcacc	
sepL+33	ggggtaccggcgaggagctgttcaccggg	
sepL+36	ggggtaccgaggagctgttcaccggggtg	
sepL+39	ggggtaccagctgttcaccggggtggtg	
sepL+42	ggggtaccgttcaccggggtggtgccc	
sepL+45	ggggtacccaccggggtggtgcccatc	
sepL+48	ggggtaccgggtggtgcccatcctg	
sepL+75	aaaaggtaccttctaattcaaaatctaatg	
sepL+78	aaaaggtaccagattctaattcaaaatcta	
sepL+81	ggggtaccaattaacgcaaaaaaattcttc	
sepL+84	ggggtaccttaacgcaaaaaaattcttctaat	
sepL+87	aaaaggtacctaattgctgagattctaatt	
sepL+90	aaaaggtacccgttaattgctgagattcta	
sepL+102	aaaaggtaccagaatttttttgcgttaattgc	
sepL+123	aaaaggtacctaatggcgaagaaatattagaag	
sepL+210	ggggtaccacctttgcgatatcccaggc	

Full-length and 51 bp translational fusions of GFP to *sepL* (pDW6 & 26) were as published previously (Table 2). Further translational fusions to the first genes of LEE1 (*ler*) and LEE5 (*tir*) were as also as published previously (Table 2). For the current study an extensive number of site-specific changes and truncations were engineered in the *sepL* transcript, based on pDW6, in order to examine the regions required for translational control (Table 2). A series of SepL-GFP fusions (Table 2) were also made for analysis of SepL translation. The primers used for the various constructs are listed in Table 3. In addition, specific changes were made in the chromosomal copy of Spot42 in the Sakai *stx*- strain by allelic exchange (Table 1). The C1–C5 and H1 changes in pDW6 were generated with the Q5 method (New England Biolabs) whilst the other mutants were generated with the Quikchange method (Agilent Technologies). All constructs were confirmed by Sanger sequencing.

### Preparation of secreted proteins and bacterial fractions for protein analyses

Bacteria were cultured in 50 ml of MEM-HEPES at 37°C (200 rpm) to an OD_600_ of 0.7–1.0. Bacterial cells were pelleted by centrifugation at 4000 *g* for 20 min, and supernatants were passed through low protein binding filters (0.45 μm). 10% TCA was used to precipitate proteins overnight, which were separated by centrifugation at 4000 *g* for 30 min at 4°C. The proteins were suspended in 150 μl of 1.5 M Tris (pH 8.8). For bacterial lysates, bacterial pellets were suspended directly in SDS PAGE loading buffer. Proteins were separated by SDS-PAGE using standard methods and Western blotting performed as described previously for EspD, RecA and EscJ (33,34). Bacteria were stained for EspA filaments following fixation with 4% paraformaldehyde for 5 min. His-EspA was purified and anti-EspA serum raised in rabbits as described (35). 1 ml of each culture was centrifuged and washed twice in PBS and the pellets were suspended in 4% PFA for 15 min. The fixed bacteria were washed twice with PBS and the pellet suspended in PBS. 20 μl was dried in triplicate on multi-spot glass slides at 42°C for 20 min. These were incubated with a 1/100 dilution of anti-EspA antibody (laboratory stocks as described above) in PBS with 0.1% bovine serum albumin (PBS-0.1% BSA) and incubated in a moist box overnight at 4°C. After three washes with PBS–0.1% BSA, the samples were incubated with fluorescein isothiocyanate-conjugated donkey anti-rabbit immunoglobulin antibody in PBS-0.1% BSA (1/500; Abcam) for 2 h in a moist box, and the slides washed three times with PBS–0.1% BSA. The slides were mounted with ProLong Gold mount medium (Thermo Fisher). The slides were then examined by confocal microscopy using a Zeiss Plan Apochromat 63×/1.40 oil immersion lens.

### *In vivo* probing of mRNA structure

The structure of *sepL* mRNA around the RBS was probed using 2-methylnicotinic acid imidazolide (NAI-N3) based on modifications of published protocols (36,37). Synthesis of NAI-N3 was performed according to (36). *Escherichia coli* were grown in LB overnight, diluted in fresh media and cultured until OD_600_ = 0.5. 25 mM NAI was added to 5 ml aliquots of the bacteria and mixed thoroughly. The mixture was incubated for 10 min at 37°C with shaking and the cells harvested by centrifugation at 4°C and flash-frozen in liquid nitrogen before storage at –80°C if not used immediately. Control and NAI-modified bacterial pellets were re-suspended in 650 μl of GTC phenol and vortexed for 3 min with 50 μl of glass beads. The suspension was incubated at 65°C for 10 min and placed on ice for another 10 min. 300 μl of chloroform/IAA was added to the suspension and then 80 μl of 100 mM NaOAc pH 5.2. The sample was vortexed and micro-centrifuged at 14 000 rpm for 5 min. The upper phase was added to 500 μl phenol:chloroform:Isoamyl alcohol (IAA) and centrifuged for 2 min at 14 000 rpm. The upper phase was removed and added to 500 μl of chloroform:IAA. The upper phase was again removed following centrifugation and added to 1 ml ethanol and 20 μg glycogen. The RNA was precipitated overnight at –20°C and pelleted by centrifugation for 30 min at 14 000 rpm and 4°C; the ethanol was removed and the pellet washed with 70% ethanol, air-dried and suspended in 50 μl MilliQ water. The RNA was measured with a Nanodrop. The primer extension was carried out using a ^32^P end-labeled primer pDW6(1)—GAATTTTTTTGCGTTAATTGCTGAG based on a published method (38). RNA was used at a concentration of 3.84 μg/μl in 1.75 μl. The extension products and sequencing ladder were run on a 6% acrylamide gel and products visualized using a FUJI-FLA 5000 imager.

### Flow cytometry

When the OD_600_ of the cultures used for population fluorescence analysis reached 0.7, bacteria were fixed by diluting a 500 μl aliquot 1:2 in 4% paraformaldehyde. Single colour fluorescence was measured using a FACScalibur flow cytometer (Becton Dickinson). Excitation was at 488 nm and emission captured at 530 nm. CELLQuest software was used to acquire and analyse flow cytometry data. A wild type culture of the strain for analysis was stained by indirect immunofluorescence for O157, using a FITC conjugated secondary antibody. This fluorescence signal was used to set the R1 gate, to exclude small particles and debris from being counted by the cytometer.

### Analysis of fluorescence levels

The total fluorescence produced by the population was determined by analyzing 150 μl aliquots of culture with a fluorescent plate reader (Fluostar Optima; BMG). Each expression experiment was carried out at least three times with separate bacterial cultures and fluorescence values were only used for analysis when the bacterial cultures were between 0.5 and 1.1 OD_600_ and these were normalized to the optical density. A student t-test was used to analyse the level of significance between strain backgrounds. For microscopy fluorescence measurements, a 50 μl aliquot was removed from the culture and diluted 1:1 in 4% PFA, and 20 μl was dried on a glass slide (37°C, 15 min) which was then washed three times with PBS and a coverslip applied using DAKO fluorescent mounting medium. Fluorescence imaging was carried out using a Leica DM LB2 microscope and a 100× objective lens. Narrow-band width filters to excite and detect eGFP/FITC were used (41017 Endow GFP, CHROMA). Images were captured using a Hamamatsu ORCA-ER black and white CCD digital camera.

### Identification of Hfq-binding sites by UV-crosslinking (CRAC analysis)

All Hfq UV-crosslinking datasets were previously described in Tree *et al.* (31) and deposited at NCBI GEO under the accession number GSE46118. Raw sequence data was aligned to the EHEC str. Sakai genome (NC_002695.1) using novoalign software and analysed using pyCRAC software (39).

## RESULTS

### Hfq interactions with the LEE determined by UV-crosslinking (CRAC)

RNAs that interact with Hfq were identified under conditions known to promote T3S expression in *E. coli* O157:H7 strains (growth in MEM-HEPES medium). Hfq UV-crosslinking (CRAC) was previously applied to *E. coli* O157:H7 strain Sakai containing chromosomally-inserted 6× His and FLAG-tagged Hfq as described (31). Under these conditions, LEE transcripts were a significant target for Hfq interactions and accounted for between 1.8–8.8% of total mapped reads in replicate Hfq-CRAC datasets (Figure 1A). In line with published research (40,41), Hfq-binding sites were recovered at both LEE1 (Ler) and within the *grlAR* di-cistronic transcript (Figure 1B,D,E). Extensive Hfq-binding was identified throughout the polycistronic LEE operons, suggesting that T3S is post-transcriptionally regulated by Hfq at many sites. Within the LEE, the LEE4 and LEE5 transcripts were the most highly recovered accounting for 27.9 ± 5.2% and 52.5 ±9.7% of reads mapping to the LEE respectively. LEE4 encodes SepL and the EspADB translocon and LEE5 encodes intimin, Tir and CesT. The LEE1 (6.2 ± 1.5%), LEE2 (9.7 ± 3.5%), LEE3 (2.3 ± 0.4%), and *grlRA* (1.2 ±1.0%) operons were recovered at lower levels, but this may reflect lower expression levels of these transcripts.

**Figure 2.**
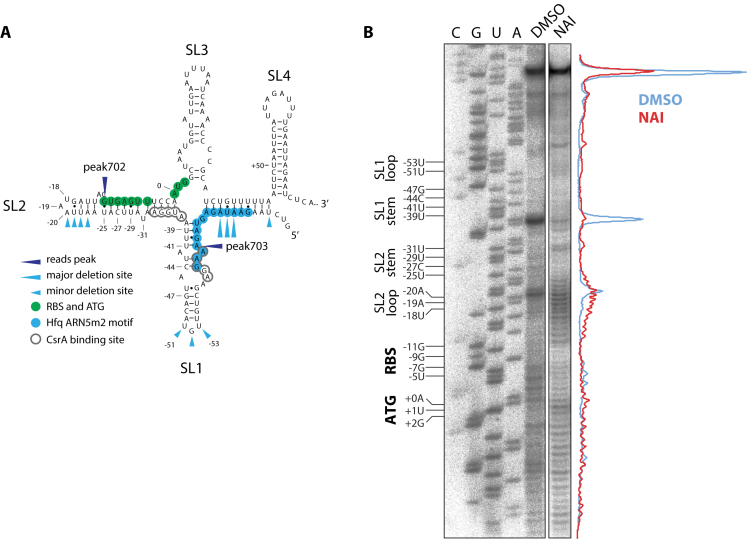
Predicted and structural analysis of the sepL 5′UTR and early coding sequence. (**A**) RNAfold prediction of the *sepL* mRNA region from –82 to +78 to highlight the predicted secondary structure around the RBS and AUG which are shown in green. Predicted CsrA binding sites are shown (27,43) along with Hfq ARN5m2 motifs and Hfq minor and major deletion sites following CRAC analysis (31). The four predicted stem loops are labelled SL1–4. (**B**) *In vivo* analysis of the RBS and AUG regions of *sepL* using SHAPE analysis. Bacteria were treated with either NAI or DMSO, RNA extracted and primer extension performed as described in Materials and Methods. The main regions of interest are indicated on the left-hand side of the figure. The traces on the right-hand side indicate radio-labeling intensity. The gels shown are representative from several analyses carried out over this region. The lack of NAI reactivity either side of stem loop 2 (SL2) supports the predicted structure (Figure 2A) with the RBS embedded within the stem region of SL2.

SepL is the product of the first gene of the polycistronic LEE4 transcript and is required for production of EspA filaments on the surface of *E. coli* O157 (8). Translational regulation of SepL would be expected to affect production of EspA filaments and T3S. The *sepL* 5′ UTR is 83nt long and is predicted to be highly structured using RNAfold (42). The 5′ UTR is predicted to fold onto the 5′ CDS to form four stem loops 11–17nt long designated SL1–4 (Figure 2A). SL2 is a 17nt stem that would sequester the ribosomal binding site (RBS). Based on this predicted structure, we hypothesized that the *sepL* mRNA would require additional factors to make the RBS accessible to the 30S ribosomal subunits and activate translation. Two principal Hfq interaction sites were mapped in the 5′ UTR of the *sepL* transcript (Figure 1B and C) and plotted on the RNAfold prediction of the *sepL* UTR (–82 to +80) (Figure 2A). A peak in Hfq-binding was identified at –80 to –50 nt from the start codon, with an associated peak in deletions (that are indicative of direct protein-RNA contact) at –65 nt (peak 703, Figure 1C). A smaller peak in Hfq-binding (peak 702) was also identified within SL2. We previously found that Hfq binds motifs consisting of five consecutive trinucleotide repeats of A, A/G, and any nucleotide, tolerating two mismatched positions (ARN5m2) in mRNAs (31). Notably, we identified an ARN5m2 site at bases –62 to –76 nt in *sepL* (highlighted blue in Figure 2A) and this motif overlaps with a potential binding site for the post-transcriptional regulator CsrA (outlined in grey in Figure 2A). Collectively, these results suggested regulatory interplay between the translationally repressive structure of the nascent *sepL* mRNA, and the RNA binding proteins Hfq and CsrA.

### Support for a sequestered RBS from structural probing of the *sepL* transcript *in vivo*

The 5′ UTR and early coding region of the *sepL* mRNA are predicted to form the structure shown in Figure 2A which includes four stem loops. According to this RNAfold structure, the *sepL* RBS is occluded in the second stem loop (SL2) which may limit translational activation unless there are further interactions to disrupt the structure allowing ribosome access to the RBS. In order to gain insights into the structure of this region of the *sepL* mRNA transcript *in vivo*, selective 2′-hydroxyl acylation (SHAPE analysis) was applied on the basis that the more flexible and often single-stranded RNA regions show more 2′-hydroxyl reactivity than RNA regions that are less flexible and usually base-paired. In our case 2-methylnicotinic acid imidazolide (NAI) was used as the electrophile. Following *in vivo* treatment of the bacteria and RNA extraction, primer extension was then used which truncates at modified bases therefore indicating where the mRNA has been processed. DMSO was used as a control treatment. This *in vivo* analysis provides support for the predicted structure, in particular the presence of stem loop 2 (SL2, Figure 2A and B), for which the two sides of the stem, including the RBS are protected from processing compared to the control and the predicted loop bases are more heavily processed (Figure 2B). The analysis also supports the prediction of the AUG codon being more accessible between two more protected regions (Figure 2B).

### An activating CsrA binding site overlaps with an Hfq interaction site in the 5′ UTR of *sepL*

**Figure 3.**
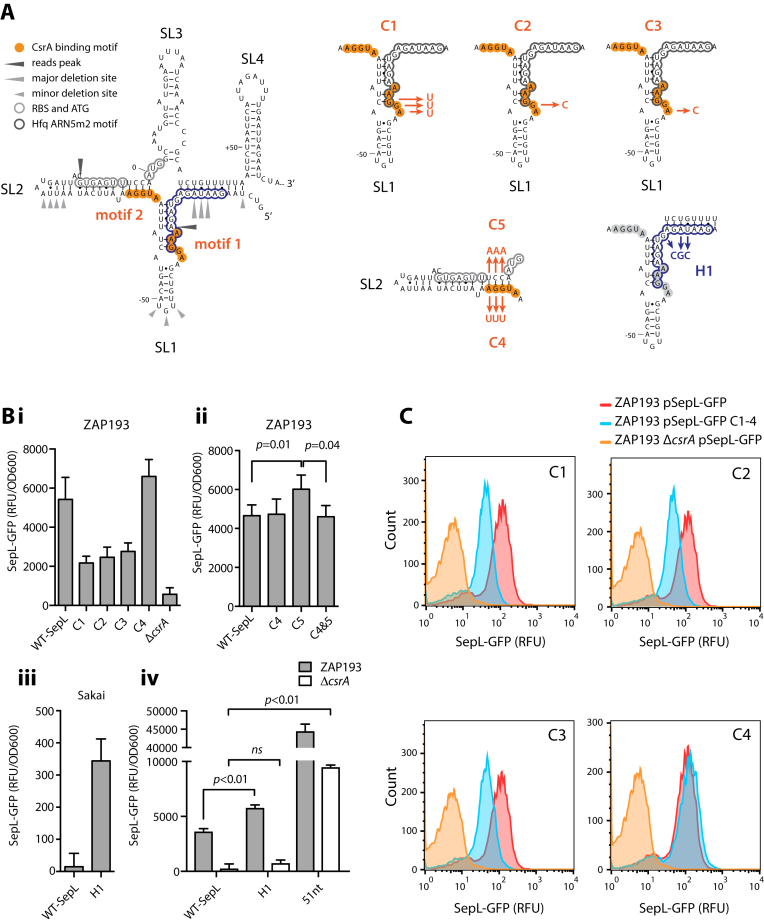
Mutagenesis of CsrA and Hfq interacting sequences in the *sepL* 5′ UTR. (**A**) the predicted secondary structure of *sepL* 5′ UTR and 5′ CDS on the left-hand side of the panel shows the two AGGnA sequences predicted to interact with CsrA. The Hfq binding peaks, deletions, and ARN5m2 motif are as indicated in Figure 1. A mutation (H1) was introduced into the ARN5m2 motif to disrupt Hfq interactions at this site (peak 703, Figure 1). Four site-specific mutations (C1–4) were introduced to test their impact on CsrA activation of *sepL* translation while C5 was introduced to restore base pairing in SL4 that may be destabilised by the C4 mutations. (**B**) Measurement of the wild type and mutated SepL-GFP fusions in either the *E. coli* O157:H7 strain ZAP193 (high T3 secretor background) or strain Sakai (low T3 secretor). Fluorescence was measured for cultures over a range of optical densities and then adjusted for optical density as described in Materials and Methods. (**C**) Flow cytometry analysis of SepL-GFP and C1–4 mutations in *E. coli* O157:H7 ZAP193 (high T3 secretor). Expression of the wild type fusion in the Δ*csrA* background indicates minimal levels of secretion in the absence of translational activation. Significance was calculated using a paired *t-*test for replicate data collected over multiple experiments (panel B, ZAP193 *sepL-*C4, -C5 and -C4&5). An unpaired *t-*test was used for all other samples.

CsrA is an activator of T3S in EPEC/EHEC and directly interacts with the 5′ UTR of the LEE4/*sepL* transcript (27). Bhatt *et al* identified two potential CsrA binding sequences that closely match the consensus AUGGA (Figure 3A, motifs 1 and 2) and demonstrated that CsrA binds the *sepL* 5′ UTR using an electrophoresis mobility shift assay (EMSA). To examine the relative contributions of each motif to *sepL* regulation, we introduced point mutations predicted to disrupt CsrA binding (Figure 3A, mutations C1–4). All point mutations were introduced into a translational reporter for *sepL* (pDW6) that includes the native promoter, the 83 nt 5′ UTR and the entire *sepL* open reading frame fused to C-terminal GFP. The WT and mutant reporters were transformed into *E. coli* O157 strain ZAP193 (NCTC12900; Table 1) that exhibits a high level of T3S expression, facilitating the measurement of any repressive effects of the mutations. A consensus motif for CsrA binding has been described (43) and so three mutations (C1–3) in motif 1 were constructed based on this consensus. C1 was predicted to have a greater effect than C2 and this in turn was predicted to have a greater impact than C3. In fact, all three markedly reduced SepL-GFP expression (*P*<0.0022) indicating a strong requirement for this consensus CsrA binding sequence (Figure 3Bi and C). While the relative levels of repression did decrease from C1 to C2 to C3, in line with their expected impact, the values were not significantly different from each other (Figure 3Bi and C).

The C4 mutation in CsrA motif 2 also destabilizes the repressive stem loop SL2 (Figure 3A) and so we also introduced a compensatory mutation C5 into SL2 to distinguish CsrA-dependent and SL2-dependent regulation. The mutation of motif 2 (C4) had little effect on SepL-GFP translation, while the C5 mutation, that changes the opposite strand to maintain predicted base-pairing, mildly increased translation (Figure 3Bii). Motif 2 is therefore unlikely to be required for CsrA-mediated translation activation. Taken together, the site-directed mutagenesis indicates that CsrA recruitment to motif 1 in SL1 is important for CsrA-dependent activation of *sepL* translation.

Since the Hfq interaction peak 703 overlaps with the motif 1 CsrA binding site, we determined whether mutation of the Hfq consensus sequence ARN5m2 altered SepL translation. The H1 mutation (Figure 3A) is predicted to remove the ARN5m2 motif without disrupting base pairing within the pitch-fork RNA structure. Introduction of mutation H1 into the translational SepL-GFP fusion increased GFP expression 1.5 fold in strain ZAP193 and 23 fold in Sakai (Figure 3Biii-iv). We conclude that the ARN5m2 motif is repressive, consistent with our Hfq-CRAC data and other Hfq binding data.

### Identification of sRNAs controlling T3S and SepL expression

**Figure 4.**
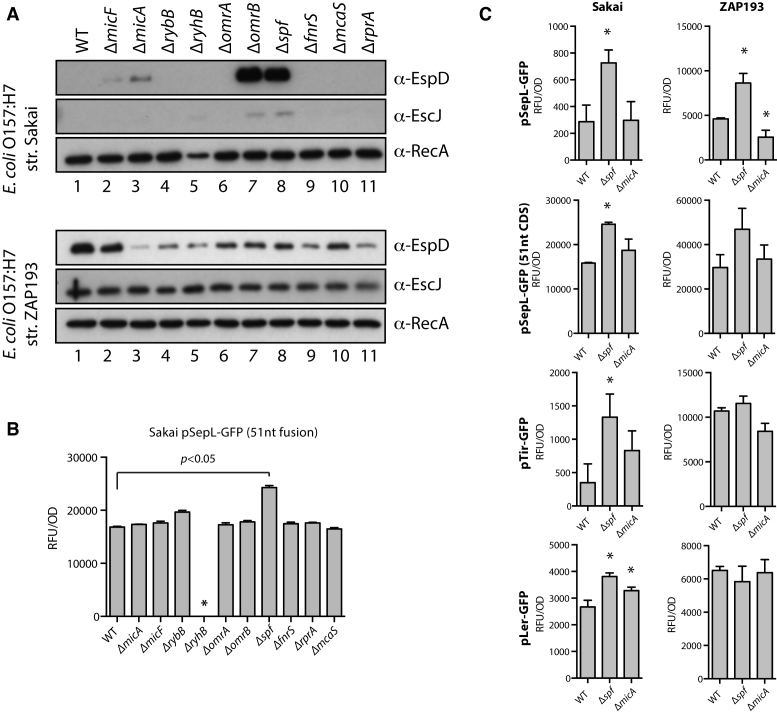
Analysis of sRNA mutations on type 3 secretion and specific LEE transcripts. (**A**) Western blot analysis for EspD (supernatant), EscJ (whole cell) and RecA (whole cell control) from WT and sRNA deletion mutants of *E. coli* O157:H7 strains Sakai (low T3 secretor) and ZAP193 (high T3 secretor). (**B**) Bar graphs showing relative expression levels of the SepL+51-GFP fusion (see text for details) in the WT and sRNA deletion backgrounds of *E. coli* O157:H7 strain Sakai. The asterisk indicates that the mutant did not grow to an OD_600_ >0.6 under our experimental conditions. (**C**) Translation of the LEE encoded proteins Ler, SepL and Tir was monitored in WT, *Δspf*, and Δ*micA* backgrounds for both low T3 secretor strain Sakai, and high T3 secretor strain ZAP193. Asterisks indicate *P*< 0.05.

We, and others, have previously shown that the sRNA binding site is closely associated with the Hfq binding site defined by CRAC or CLIP-Seq (31,44). We therefore restricted our search for complementary sRNAs to the Hfq binding site defined by Hfq-CRAC reads in *sepL* (bases –82 to +50; Figure 1C) using the IntaRNA program (45). We focused our search to sRNAs that were abundant in our Hfq-CRAC data, which were: MicA, MicF, RybB, RyhB, OmrA, OmrB, FnrS, Spot42, McaS and RprA. Of these sRNAs, MicF, FnrS, Spot42, and OmrA had some complementarity to the *sepL* Hfq-binding site. Each of the ten sRNAs were deleted in ZAP193 (high T3S) and Sakai (low T3S), to allow the assessment of negative and positive regulatory effects, respectively (Figure 4A). These strains were analysed for secreted EspD in bacterial supernatants as an indicator of T3S and translocon expression. EscJ, a basal T3S apparatus protein and RecA were assayed from the associated whole cell pellets as controls for T3S basal apparatus expression and culture density respectively (Figure 4A). Deletion of either *omrB* or *spf* (Spot42) was found to increase secreted levels of EspD and total levels of EscJ.

The effects of these sRNA on SepL translation were assayed using a truncated *sepL* fusion containing the 5′ UTR and 51 nt of the CDS fused at the C-terminal to GFP (pDW26, Table 2). We had previously found that this truncated *sepL* fusion was translated at high levels in all cells, especially in strain Sakai (low T3S), potentially reflecting the absence of repressive structure or sequence demonstrated for the full-length transcript (see final results section). This allows any repression by the sRNAs in strain Sakai to be assessed as the transcript is now being effectively translated (Figure 4B). The expression of the truncated SepL-GFP fusion was clearly increased only by deletion of *spf* (encoding Spot42), indicating that Spot42 negatively regulates SepL translation. The sRNA deletions were combined with additional GFP reporters in both ZAP193 and Sakai backgrounds to measure effects on expression of other T3S system genes. These included translational reporters to Ler (pLer-GFP; first gene of LEE1), the translocated intimin receptor Tir (pTir-GFP; first gene in LEE5), full length SepL (pSepL-GFP; LEE4) and the truncated *sepL* fusion containing the 5′ UTR and 51nt of CDS (pDW26) (Figure 4B). Measuring GFP expression throughout the growth of the bacterial cultures showed that the Spot42 deletion (Δ*spf*) increased the expression of both SepL fusions in either strain. This was confirmed by analysis of optical density-adjusted readings for the translational fusions between OD_600_ 0.6 and 1.0 (Figure 4B and C), an optimal period of T3S expression under these culture conditions. In addition, Δ*spf* was associated with increased GFP expression from the Tir and Ler translational fusions, especially in the Sakai background (Figure 4C). A *micA* deletion was also analysed in more detail in the two backgrounds as there was some evidence of regulation by this sRNA based on the EspD and EspJ secretion profiles (Figure 4A). However, there was no consistent pattern of altered GFP expression for this sRNA and it was not studied further (Figure 4C). OmrB was also excluded from further analyses as there was no evidence from the translational reporters for regulation of SepL, Tir or Ler despite the effect of the mutation on T3S, suggesting these affects are indirect (Figure 4B and data not shown). Cumulatively, these results demonstrate that Spot42 represses T3S and negatively affects expression of Ler, Tir and SepL.

### Spot42 directly represses SepL translation

**Figure 5.**
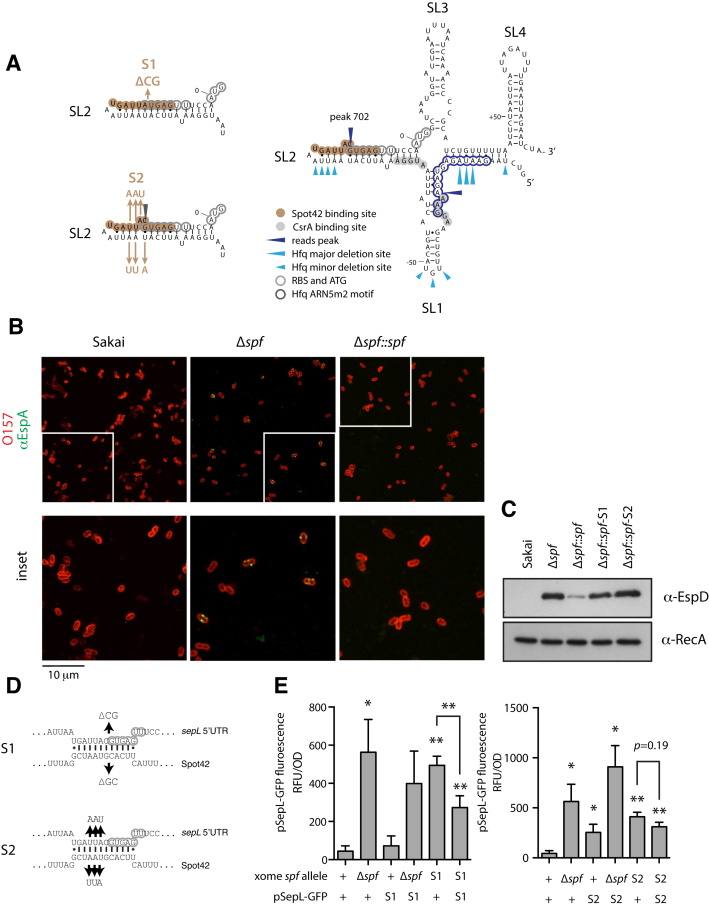
Analysis of Hfq and Spot42 interactions with the *sepL* transcript. (**A**) Two site specific mutations in predicted stem loop (SL) 2 are shown (S1 and S2) along with other features described in Figures 1 and 2. (**B**) EspA filament staining on the surface of *E. coli* O157:H7 strain Sakai and the isogenic Δ*spf* and complemented strains. EspA filament staining (green) was carried out as described in Materials and Methods with the bacteria surface stained using anti-O157 (red). (**C**) Western blot for EspD in bacterial supernatants and RecA from whole cell preparations from WT strain Sakai, the Δ*spf* deletion mutant, and chromosomal knock-ins using *spf*, the *spf-S1*, or *spf-S2*, to replace the deleted region. (**D**) Predicted base pairing between *sepL* and Spot42, and compensatory mutations in the *sepL* 5′ UTR predicted to restore base pairing with the *spf-S1* and *spf-S2* site-directed mutants. (**E**) Plasmid-based SepL-GFP fusions carrying the S1 and S2 mutations were assayed in wild type *E. coli* O157:H7 strain Sakai, the isogenic *spf* deletion, and chromosomal knock-ins of the *spf-S1* and *spf-S2* mutations (indicated below). Significance was calculated relative to WT *spf* and pSepL-GFP fluorescence unless indicated. A single asterisk indicates *P*≤ 0.1 and a double asterisk indicates *P*≤ 0.05.

The polycistronic LEE4 operon encodes the filament protein EspA and pore forming proteins, EspBD. Repression of *sepL* translation by Spot42 binding in SL2 (Figure 5A) is predicted to block filament expression and transport to the cell surface. As further confirmation of regulation by Spot42, we used immunofluorescence to monitor EspA filament expression in the low-secretor strain Sakai, an isogenic Δ*spf* variant, and its chromosomally-repaired complement (Δ*spf*::*spf*). Deletion of *spf* increased the frequency of EspA filamentation on bacterial cells, whereas filamentation was reduced to wild type levels in the chromosomally repaired strain (Figure 5B). This result was supported by western blotting for EspD in the *Δspf* and complemented backgrounds (Figure 5C).

Our data indicated that Spot42 acts on several operons contributing to T3S, including LEE1, and we therefore determined whether Spot42 directly regulates *sepL* translation. Spot42 was predicted to interact with the LEE4 transcript adjacent to the *sepL* ribosomal binding site, at peak 702 (Figure 5A and D). Two mutations (S1 and S2, Figure 5A) were constructed in the full length SepL translational fusion and transformed into Sakai to monitor potential de-repression of T3S. As SL2 is predicted to form a repressive stem loop at the RBS, mutation S2 was designed to retain base pairing of this structure with reciprocal changes introduced on the opposite side of the stem loop (Figure 5A). The S1 mutation did not result in a significant increase in expression in the wild type background, while the S2 mutation increased expression about five-fold *(P* < 0.05), which is ∼50% of the increase in expression observed for the *spf* deletion under these conditions (Figure 5E).

Two compensatory mutations were then introduced into the chromosomal copy of *spf* to match the changes made in the proposed Spot42 binding site (*sepL-S1* and -*S2*, Figure 5D). Both of the chromosomal *spf-S1* and *spf-S2* point mutations increased T3S to levels comparable to the Δ*spf* strain (Figure 5C), indicating that these chromosomal *spf* mutations act in an equivalent way to *spf* deletion. Similarly, when the strains expressing the *spf-S1* or *spf-S2* chromosomal alleles were transformed with the wild type SepL-GFP fusion, GFP expression was de-repressed to levels comparable to the Δ*spf* mutant (Figure 5E). When the reciprocally modified SepL-GFP fusions were introduced into the Δ*spf* strain it was noted that *sepL-S2* trended towards higher expression (*P =* 0.27) suggesting that part of this increased activity may be *spf* independent. When both fusions were then matched with their cognate *spf-S1* or -*S2* backgrounds, both showed reduced expression (Figure 5E). This was statistically significant for the *sepL-S1* mutant (*P =* 0.05) relative to the wild type *sepL* fusion in this background, and trending downward for the *sepL-S2* mutant (*P =* 0.19) relative to the wild type *sepL* fusion in the *spf-S2* background. Cumulatively, these data demonstrate that Spot42 base pairs with the *sepL* 5′ UTR directly adjacent to the ribosomal binding site.

### Sequence requirements for SepL translation

**Figure 6.**
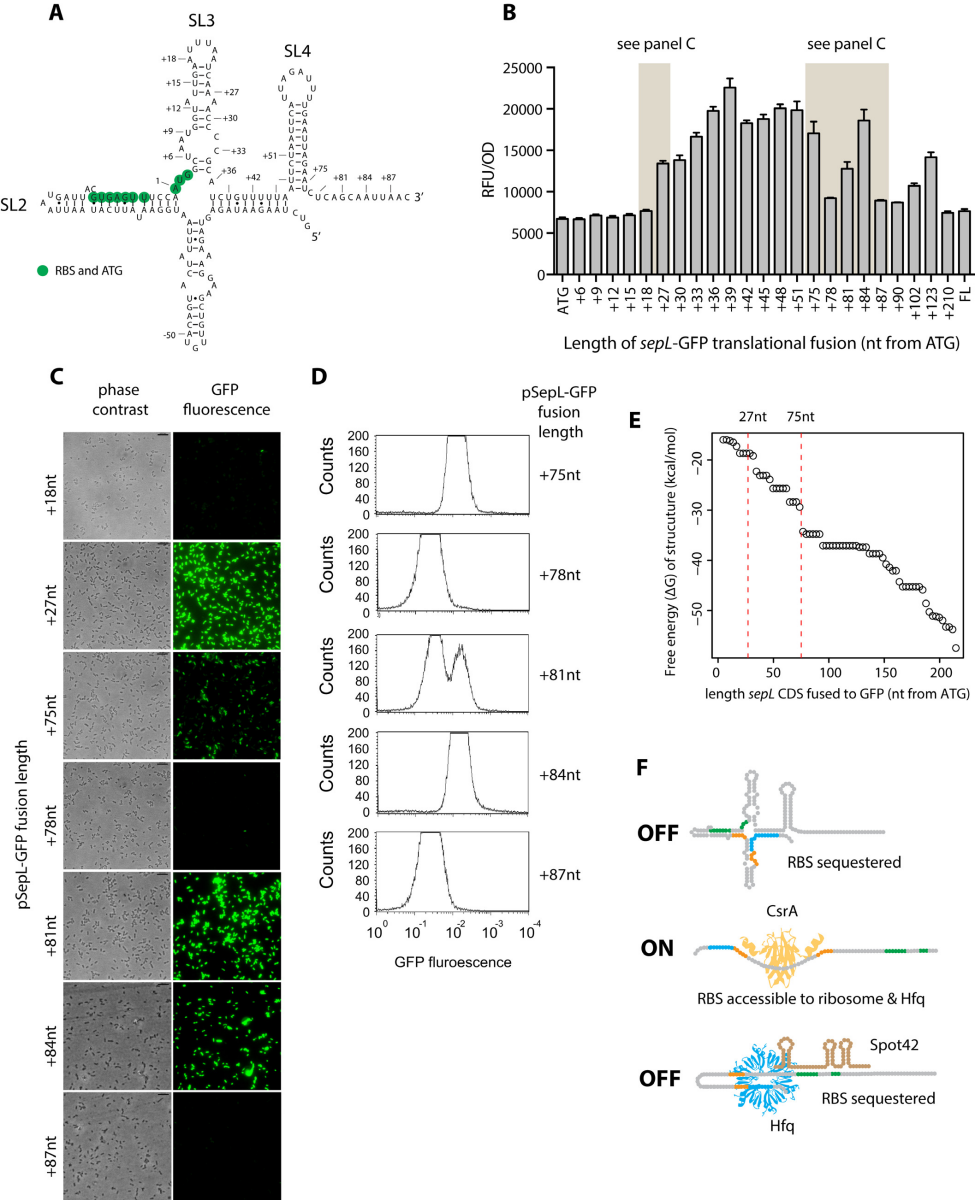
SepL translation and summary model. (**A**) The predicted RNAfold structure of the 5′ UTR and first 89 nt of *sepL* CDS is shown for orientation of possible secondary structure absent in *sepL* truncates shown in panel B. (**B**) A systematic series of fusions to GFP were constructed into the *sepL* open reading frame to investigate sequence requirements for expression. All the fusions included the 5′ UTR and natural promoter of *sepL* (Table 2). The shortest fusion encompassed the native *sepL* promoter to the initiation codon (ATG). Successive three nucleotide extensions are numbered by how far they extend (in bases) into *sepL*. The final construct is the full length (FL) fusion used in previous studies (pDW6, Table 2). Expression from this series of constructs was measured in the low secretor strain Sakai. (**C**) Fluorescence microscopy of selected *sepL’*-GFP constructs to illustrate transitions in *sepL* translation. The 81 nt fusion exhibits a clear biphasic expression pattern, with approximately half of the cells expressing GFP. (**D**) Flow cytometry of *sepL* truncates presented in panel C. (**E**) Graph showing the maximum ΔG values for the different truncates fused to eGFP. This provides an indication of when major transitions in structure may occur between the different length fusions. (**F**) Model of the post-transcriptional regulation leading to transient expression of the *sepL* transcript. The model is described within the Discussion section of the manuscript. It is proposed that the transcript adopts a structure that initially prevents translation due to poor access to the RBS. CsrA is required for translational activation along with specific sequence in the early part of *sepL* (1–18 bases). SepL translation is silenced by Hfq-Spot42 binding to occlude the RBS and potentially by direct competition with CsrA. This dynamic is proposed to result in transient SepL translation and assembly of the EspA translocon.

To identify additional sequence requirements for translational control of SepL, a series of GFP fusions were constructed at different positions in the *sepL* CDS (Figure 6A–B). All included the 83 base 5′ UTR and native promoter of *sepL* (Table 2). GFP expression was measured in strain Sakai (Figure 6B–D). There was little detectable translation until the first 27 nucleotides of the open reading frame were included in the construct, indicating the presence of 5′ elements essential for translation, potentially through formation of predicted stem loop SL3 (Figure 6B). Expression increased for the fusion at nt +27 and remained relatively high until nt +75, for which expression was significantly lower. GFP levels increased again for fusions at nt +81 and nt +84, after which expression was reduced and remained relatively low and equivalent to the full-length construct (FL, Figure 6A–D). ΔG values based on RNAfold modelling of the different length *sepL* truncates fused to eGFP were plotted (Figure 6E) and this predicts key transitions in structure at ∼27nt and ∼75nt (Figure 6E). While these are only estimations, the latter transition may correlate with variability in expression measured for constructs from bases +75 to +87, (Figure 6A–D). Specifically, the +81 nt construct exhibited a biphasic expression pattern representing instability between high- and low-level expression (Figure 6E). The transitions in GFP expression measured in Figure 6B correlated well with the positions of stem loops SL3 and SL4 in the predicted structure (Figure 6A), although complete deletion of the sequence within the predicted stem loop SL4 had no effect on expression (data not shown). In summary, the different length SepL-GFP constructs provide evidence for a minimum sequence requirement to initiate translation, with a longer sequence required for default repression of the *sepL* transcript.

## DISCUSSION

T3S in *E. coli* O157 is subject to complex regulation as confirmed by the significant number of regulators that act on the system and marked variation in the secretion phenotype between isolates (4,46). This variability reflects multiple regulatory inputs at both transcriptional and post-transcriptional levels. We previously reported that EspA filaments, expressed from the LEE4 operon, are controlled post-transcriptionally but the basis for this was not known (25,26). Subsequently, the sRNA chaperone, Hfq, was shown to repress T3S in two studies (40,41) although a third showed activation (47). The main proposed sites for this control were Ler and GrlA/R, both key early regulators of T3S. Further post-transcriptional regulation of the LEE was identified by Bhatt *et al.* (27), demonstrating that CsrA negatively regulated GrlRA expression and positively regulated LEE4 at the *sepL* 5′UTR.

The present study extends our genome-wide approach (31) to analyse the sequences interacting with Hfq under culture conditions in which the T3S system is expressed, allowing the mapping of Hfq binding across the locus of enterocyte effacement operons (LEE1–5). CRAC UV-crosslinking of Hfq confirmed previously proposed targets for regulation by Hfq adjacent to *ler* (LEE1) and *grlRA*. In addition, we identified extensive Hfq binding to LEE4 and LEE5 mRNAs, consistent with our previous report of coupled post-transcriptional regulation of these two operons (26). The CRAC data defined two peaks of Hfq binding in the *sepL* 5′ UTR (peaks 703 and 702, Figure 1), with peak 703 containing a cannonical ARN motif recognized by the distal face of Hfq. This recruits Spot42 to the *sepL* seed motif 43nt downstream to occlude translation initiation. Mutational analysis also confirmed the primary AUGGA motif required for CsrA activation, which overlaps with the second mapped Hfq interaction sequence (peak 703) suggesting Hfq and CrsA binding is mutually exclusive.

We propose that the combination of activation by CsrA followed by Hfq-mediated Spot42 repression are components of an autonomous OFF-ON-OFF switch or ‘toggle’ that results in transient SepL translation (Figure 6F). Under the model it is assumed that SepL translation is a pre-requisite for EspA filament production, as demonstrated by others (10,30). Our *in vivo* structural probing of the *sepL* transcript indicates that the default structure formed correlates well with that based on free energy modelling (shown in Figures 2-6). In this structure, the Shine Dalgarno (SD) is sequestered in a stem and the start codon is protected at the base of this stem loop. We conclude that translation would initially be repressed following transcription based on structural constraints imposed by the mRNA structure as indicated in this study and depicted in our model (Figure 6F). Multiple regulatory factors may contribute to translation of *sepL* and here we demonstrate that CsrA binding to SL1 is required for translation activation, most likely by opening the repressive SL2 structure to allow ribosome access to the SD. Additional factors may be required for this activation which would help explain heterogeneity in SepL and EspA expression at the single cell level observed in this and other studies (Figures 5B and 6C–D) (25,26).

Our analysis then demonstrates that translation of SepL is repressed by Spot42 chaperoned by Hfq. Spot42 is present at high levels under conditions in which cAMP concentrations are low, as *spf* (encoding Spot42) is repressed by CRP when complexed with cAMP (48–50). Therefore, Spot42 is also present in bacterial cells with an overall positive energetic state, along with CsrA. A regulatory circuit that juxtaposes CsrA and Spot42 function would then be expected to rapidly transition between ON and OFF states. We therefore propose they are part of an ON-OFF regulatory circuit acting at the translational level and this is likely to result in transient expression of SepL at the single cell level (Figure 6F). We note that a tipping point between repression and translation was identified in our 81nt translational fusion which we presume represents the mRNA oscillating between closed and open structures producing a heterogeneous population in terms of GFP expression (Figure. 6C–E). This transition point matched closely with RNAfold predictions of conformations for the different modelled fusion transcripts and their respective free energies (Figure 6E). Future work will address the full structure of the mRNA and any additional factors needed to alter the stability of the stem loops demonstrated in the 5′UTR and early part of the reading frame.

Our study, building on the previous work of Bhatt *et al.* (27), shows that CsrA is essential for translation and CsrA is likely to work in a manner similar to MoaA activation in *E. coli* (51) and Phz2 activation in *Pseudomonas* (52) by opening or maintaining a translationally active conformation of the transcript (53). The fact that Spot42 repressed translation of both the full length SepL-GFP fusion and the shorter (51 base/17aa) fusion (Figure 3B–C), which shows high levels of expression and is predicted to represent the open structure, supports the idea that Hfq-Spot42 act on a translationally ‘ON’ *sepL* mRNA. Biophysical characterization of Hfq-mRNA interactions indicate a dissociation constant (*K*_d_) of 1–4 nM, and CsrA has been shown to bind the *sepL* 5′UTR with a K_d_ of 23nM (27) suggesting that Hfq repression would be favoured once access to the single-stranded site is available.

We had previously found that Hfq- and sRNA-binding sites were closely associated in mRNAs, potentially to facilitate dissociation of mRNA–sRNA duplexes, but also to position the mRNA seed for annealing at the lateral surface of Hfq (31,54,55). The distal face of Hfq can accommodate 18nt of RNA, and a linear spacer of 43nt before the Spot42 binding site would position the mRNA seed far from the lateral edge of Hfq. For *sepL*, it appears that extensive secondary structure may allow longer range contacts with Hfq and a second ‘ARN-less’ Hfq binding peak (702) at the Spot42 binding site indicating that this distant site is drawn in close enough to make contact with Hfq.

Hfq was also found to bind at multiple sites throughout the polycistronic LEE5 operon (that encodes *tir-cesT-eae*), with the most prominent binding sites occurring within the upstream *map* 5′UTR, within the *tir* CDS, and at the 3′ end of *eae*. We found that Spot42 regulates the expression of Tir in the low secretor Sakai background, indicating multiple points of Spot42-dependent regulation. Recent work in *Vibrio parahaemolyticus* has demonstrated Spot42 regulation of the CesT-family protein VP1680 (56) and it appears that post-transcriptional Spot42 regulation of this effector chaperone is likely conserved in EHEC. The LEE4 transcript also contained extensive sites of Hfq-binding with prominent peaks within the CDS of *espD, espB*, and the 3′ of *orf29*. The latter is in good agreement with the binding site identified for GlmZ (57) that is on the 5′ edge of this Hfq peak. Interestingly, RNA-RNA interactions 5′ of the Hfq binding site is more consistent with an sRNA than an mRNA (44). The arrangement of Hfq and sRNA binding sites at the 3′ of *orf29* suggests that this site may represent a 3′ UTR sRNA and is reminiscent of the sRNA-sponge, SroC, that is generated from the 3′ of *gltI* to sponge the sRNA GcvB (58). The precise mechanism of GlmZ-*orf29* regulation of LEE4 is currently unclear, but our Hfq-binding data confirms that this is a site of Hfq-dependent regulation and suggests a 3′UTR sRNA may be involved.

In the present study we focused on strain Sakai, a low secretor, which reflects the secretion state of many wild type strains we have examined (26). Our proposed model is based on Hfq interactions measured in this strain and mutations studied in different Sakai genetic backgrounds. However, the high secretor strain (ZAP193) was also included in the current study, and while SepL translation is repressed by Spot42 in this background, there was no evidence that this led to increased EspADB translocon production in the Δ*spf* background. We consider that this indicates export control of the translocon apparatus is saturated in this high secretor background and so de-repressing SepL levels has no impact on filament production under these conditions.

In summary, CsrA and Spot42 are used to create a post-transcriptional toggle that we predict will lead to a burst of translation that is rapidly silenced. The ON-OFF kinetics would be tuned by the concentrations of free CsrA, and Hfq-Spot42. Spot42 is an early bacterial sRNA in evolutionarily terms and is expressed at high levels and so we anticipate that this simple post-transcriptional toggle would be a useful regulatory motif for a range of proteins required transiently in limited quantities.
